# Racial and Ethnic Disparities in COVID-19 Mortality

**DOI:** 10.1001/jamanetworkopen.2024.11656

**Published:** 2024-05-21

**Authors:** Jake Ryann C. Sumibcay, Dennis Kunichoff, Mary T. Bassett

**Affiliations:** 1 FXB Center for Health and Human Rights, Harvard T.H. Chan School of Public Health, Boston, Massachusetts; 2 Department of Health Policy and Management, Harvard T.H. Chan School of Public Health, Boston, Massachusetts

## Abstract

This cross-sectional study examines racial and ethnic differences in COVID-19 mortality in the United States across 4 case surges between February 2020 and September 2023.

## Introduction

In May 2023, the United States ended the federal COVID-19 Public Health Emergency (PHE) with more than 1.1 million reported deaths.^1^ American Indian or Alaska Native and Native Hawaiian or Pacific Islander individuals represent numerically small populations (constituting 1.3% and 0.3% of the total US population,^2^ respectively). Their high mortality rates have minimal impact on national rates and may receive little attention.^3^ To uncover these disparities, we analyzed separately the racial and ethnic mortality trends during the PHE, focusing on surge periods.

## Methods

This cross-sectional analysis used publicly available COVID-19 mortality data from the National Center for Health Statistics^4^ from February 2020 to September 2023 for 6 defined racial and ethnic groups: Hispanic, non-Hispanic American Indian or Alaska Native, non-Hispanic Asian, non-Hispanic Black, non-Hispanic Native Hawaiian or Pacific Islander, and non-Hispanic White. We added an additional category combining non-Hispanic Asian and non-Hispanic Native Hawaiian or Pacific Islander to examine the effect of the historical Asian–Pacific Islander grouping.^3^ Cumulative age-adjusted mortality rates per 100 000 individuals were calculated for each racial group over 4 periods (February to June 2020, October 2020 to March 2021, June to October 2021, and November 2021 to March 2022), selected to correspond to the surges of COVID-19 mortality. We computed rate ratios with 95% CIs for each specified period, using non-Hispanic White individuals as the reference group. All analyses were performed using R version 4.2.2 (R Core Team 2022). Additional details are provided in the eMethods in Supplement 1. This study was deemed not human participant research by the Harvard Longwood Campus institutional review board at Harvard University; therefore, the requirement for informed consent was waived. We followed the STROBE reporting guidelines.

## Results

During the examined period, the number of COVID-19 deaths equaled 172 129 for Hispanic individuals, 12 113 for non-Hispanic American Indian or Alaska Native individuals, 35 392 for non-Hispanic Asian individuals, 157 072 for non-Hispanic Black individuals, 2321 for non-Hispanic Native Hawaiian or Pacific Islander individuals, and 758 221 for non-Hispanic White individuals; the corresponding population sizes were 60.6 million, 2.4 million, 19.7 million, 41.9 million, 0.6 million, and 196.8 million, respectively (Table). Non-Hispanic Black, Hispanic, non-Hispanic American Indian or Alaska Native, and non-Hispanic Native Hawaiian or Pacific Islander groups experienced higher mortality than non-Hispanic White individuals, with disparities widening during surges. Non-Hispanic American Indian or Alaska Native and non-Hispanic Native Hawaiian or Pacific Islander groups consistently exhibited higher mortality rates compared with all other racial and ethnic groups during the second, third, and fourth periods (Figure). Throughout, non-Hispanic American Indian or Alaska Native individuals experienced the highest mortality rates, and Hispanic, non-Hispanic Native Hawaiian or Pacific Islander, and non-Hispanic Black populations followed closely (Table). Non-Hispanic Native Hawaiian or Pacific Islander individuals had the highest mortality rate among all groups during the third time interval, yielding the largest rate ratio when compared with non-Hispanic White individuals (Table). In contrast, non-Hispanic Asian individuals displayed the lowest mortality rates during the second, third, and fourth periods, with the second-lowest rates during the first period (Table). Non-Hispanic Asian and non-Hispanic Native Hawaiian or Pacific Islander groups combined exhibited similar results to the non-Hispanic Asian group alone (Table).

**Table.** Age-Adjusted COVID-19 Mortality Rates and Rate Ratios by Race and Ethnicity Examined From February 2020 to September 2023 and at 4 Periods, Corresponding to Surges of COVID-19

Race	2020-2021 Population estimates, No.	Entire period			First surge		Second surge		Third surge		Fourth surge	
		February 2020 to September 2023			February to June 2020		October 2020 to March 2021		June to October 2021		November 2021 to March 2022	
		Total deaths, No.	Age-adjusted mortality rates (95% CI)	Rate ratios (95% CI)	Age-adjusted mortality rates (95% CI)	Rate ratios (95% CI)	Age-adjusted mortality rates (95% CI)	Rate ratios (95% CI)	Age-adjusted mortality rates (95% CI)	Rate ratios (95% CI)	Age-adjusted mortality rates (95% CI)	Rate ratios (95% CI)
Hispanic	60 647 504	172 129	406.1 (404.1-408.1)	1.56 (1.38-1.76)	58.9 (58.1-59.7)	2.55 (1.70-3.83)	143.4 (142.3-144.6)	1.82 (1.46-2.27)	54.1 (53.4-54.8)	1.26 (0.93-1.69)	61.5 (60.7-62.3)	1.11 (0.86-1.45)
Non-Hispanic American Indian or Alaska Native	2 451 916	12 113	495.0 (486.0-504.2)	1.90 (1.68-2.15)	51.3 (48.3-54.3)	2.22 (1.47-3.35)	175.2 (169.9-180.7)	2.23 (1.78-2.78)	80.0 (76.4-83.7)	1.86 (1.37-2.51)	95.4 (91.5-99.4)	1.73 (1.32-2.26)
Non-Hispanic Asian	19 685 901	35 932	178.1 (176.2-180.0)	0.69 (0.61-0.77)	32.9 (32.1-33.7)	1.42 (0.95-2.14)	65.4 (64.2-66.5)	0.83 (0.67-1.04)	15.2 (14.6-15.7)	0.35 (0.26-0.48)	25.5 (24.8-26.2)	0.46 (0.35-0.60)
Non-Hispanic Black	41 858 536	157 072	396.7 (394.7-398.7)	1.53 (1.35-1.72)	80.2 (79.3-81.1)	3.47 (2.31-5.22)	102.8 (101.8-103.8)	1.31 (1.05-1.63)	62.0 (61.2-62.7)	1.44 (1.07-1.94)	66.5 (65.7-67.4)	1.20 (0.93-1.57)
Non-Hispanic Native Hawaiian or Pacific Islander	626 246	2321	403.6 (386.9-420.8)	1.55 (1.37-1.77)	29.1 (24.7-34.1)	1.26 (0.81-1.95)	112.0 (103.1-121.4)	1.42 (1.13-1.80)	105.4 (97.2-114.2)	2.45 (1.8-3.33)	74.1 (67.0-81.8)	1.34 (1.01-1.78)
Non-Hispanic Native Hawaiian or Pacific Islander and Asian	20 312 147	38 253	184.4 (182.6-186.3)	0.71 (0.63-0.80)	32.9 (32.1-33.7)	1.42 (0.95-2.14)	66.7 (65.6-67.8)	0.85 (0.68-1.06)	17.6 (17.1-18.2)	0.41 (0.3-0.55)	26.8 (26.1-27.5)	0.49 (0.37-0.63)
Non-Hispanic White	196 833 431	758 221	259.9 (259.3-260.5)	1 [Reference]	23.1 (22.9-23.3)	1 [Reference]	78.7 (78.4-79.1)	1 [Reference]	43.1 (42.8-43.3)	1 [Reference]	55.2 (54.9-55.5)	1 [Reference]

**Figure.**
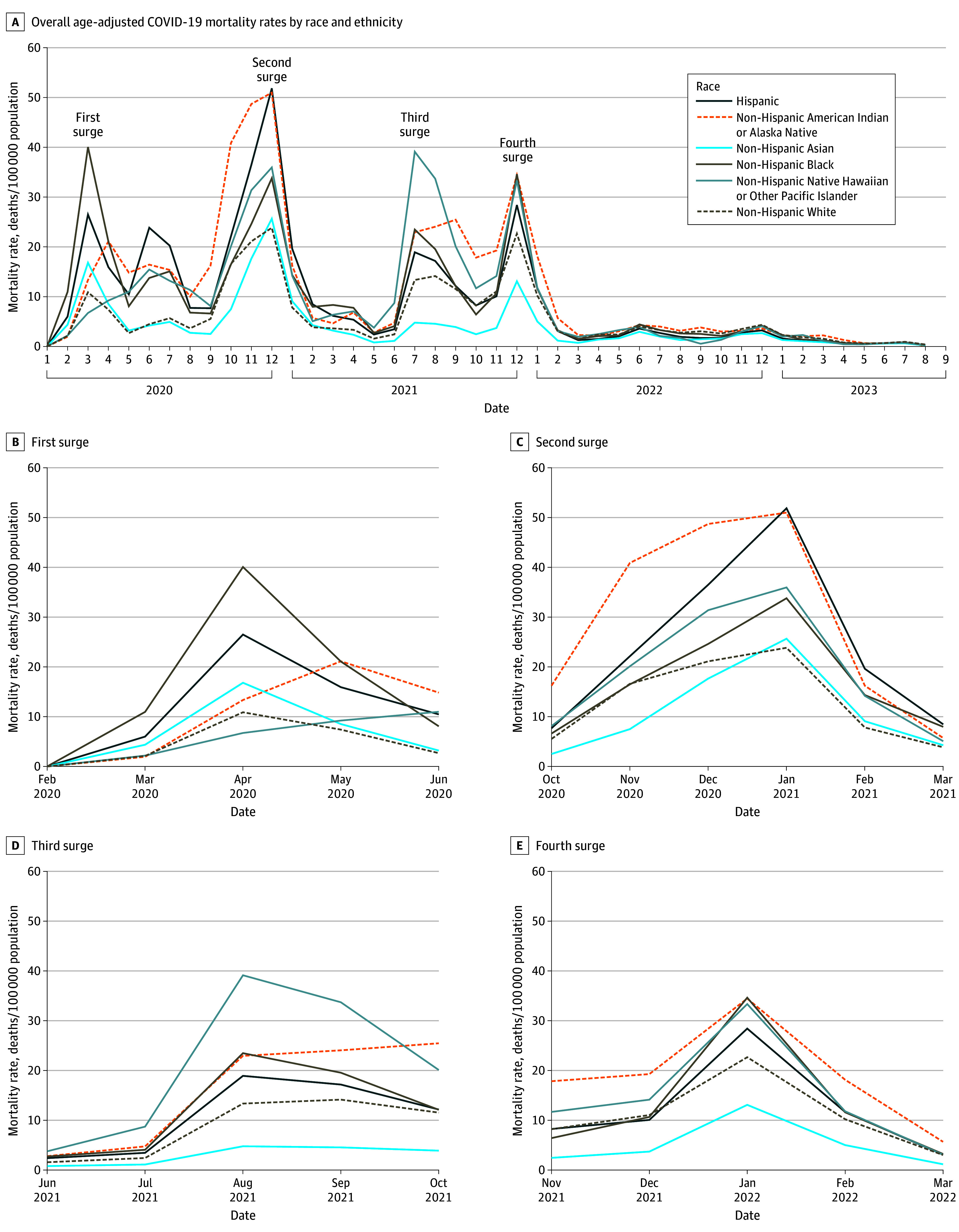
Age-Adjusted COVID-19 Mortality Rate (per 100 000 Population) by Race and Ethnicity Examined at 4 Periods, Corresponding to Surges of COVID-19 Public Health Emergency First surge was defined as February to June 2020; second surge, October 2020 to March 2021; third surge, June to October 2021; fourth surge, November 2021 to March 2022.

## Discussion

This analysis underscores the persistently high mortality risk for American Indian or Alaska Native and Native Hawaiian or Pacific Islander individuals during the PHE, exacerbated by the emergence of new variants of COVID-19. Limitations, such as data gaps, underreporting, and the misclassification of race and ethnicity (eg, death records), may lead to underestimating rates for specific groups like American Indian or Alaska Native, Native Hawaiian or Pacific Islander, and multiracial populations.^5^ Encouraging consistent use and examination of racial and ethnic disaggregated data are critical to elucidating disparities. Combining Native Hawaiian or Pacific Islander individuals with Asian individuals, a group with lower mortality, masked Native Hawaiian or Pacific Islander persistent mortality risk, cautioning against the Asian–Pacific Islander categorization. The absence of further granular data limited the assessment of within-group differences. Aggregated data hamper the identification of crucial patterns, hindering the development of targeted interventions, such as financial resources and community-driven, culturally sensitive initiatives. It is noteworthy that 2 groups (American Indian or Alaska Native, Native Hawaiian or Pacific Islander) with persistently high COVID-19 mortality represent Indigenous populations subjected to racism and colonialism, suggesting the influence of historical trauma and genocidal policies.^6^ Investigating the unique and upstream factors influencing mortality is critical to producing accurate epidemiological accounts of smaller groups with greater risk.

## Notes

Supplement 1. **eMethods.** Supplementary Methods

Supplement 2.
Data Sharing Statement
