# Supplementary material for: Racial and Ethnic Disparities in COVID-19 Mortality
Source: JAMA Netw Open. 2024 May 21;7(5):e2411656. doi: 10.1001/jamanetworkopen.2024.11656 (PMC11109770; doi:10.1001/jamanetworkopen.2024.11656)
Supplement: Supplement 1. — eMethods. Supplementary Methods [file jamanetwopen-e2411656-s001.pdf]

Supplemental Online Content

Sumibcay JC, Kunichoff D, Bassett MT. Racial and Ethnic Disparities in COVID-19 Mortality. *JAMA Netw Open.* 2024;7(5):e2411656.
doi:10.1001/jamanetworkopen.2024.11656

eMethods. Supplementary Methods

This supplemental material has been provided by the authors to give readers additional information about their work.

© 2024 Sumibcay JC et al. *JAMA Network Open.*

eMethods. Supplementary Methods

We used all available weekly COVID-19 mortality data published by the National Center for Health Statistics (NCHS) derived from death certificate information reported directly to the NCHS, which processes, codes, and tabulates the data. We used data collected from February 2020 to September 2023, stratified by the six defined racial and ethnic groups (Hispanic, non-Hispanic American Indian or Alaska Native, non-Hispanic Asian, non-Hispanic Black, non-Hispanic Native Hawaiian or Pacific Islander, and non-Hispanic White) and 9 age categories (defined below), and aggregated the weekly mortality counts into monthly counts.

We used US Census Single-Race Population Estimates for 2020-2021 to first calculate race-specific mortality rates for each age group, and then we used the Year 2000 Standard Population for the United States to calculate the age-adjusted mortality rates per 100,000 individuals.

We also computed rate-ratios with 95% confidence intervals for the entire time range in aggregate and each of the defined time periods (February 2020 to June 2020, October 2020 to March 2021, June 2021 to October 2021, and November 2021 to March 2022), using NH White as the reference group for the rate-ratios. We used the following formula to compute the direct age-standardized rates.[1]

$$(R(std) = \Sigma I \ (Psi/ Ps) \ Ri)$$

Where ***Psi*** is the standard population for the age group ***i*** (using 9 age categories: 0-4, 5-17, 18-29, 30-39, 40-49, 50-64, 65-74, 75-84, 85+ years old), ***Ps*** is the total US standard population (all ages combined), and ***Ri*** is the age-specific death rate for the age group ***i.***

All analyses were performed using R Statistical Software (v4.2.2; R Core Team 2022).

Reference

1. https://wonder.cdc.gov/wonder/help/ucd.html#2000%20Standard%20Population

© 2024 Sumibcay JC et al. *JAMA Network Open.*
