# Supplementary material for: Racial and Ethnic Disparities in COVID-19 Mortality
Source: JAMA Netw Open. 2024 May 21;7(5):e2411656. doi: 10.1001/jamanetworkopen.2024.11656 (PMC11109770; doi:10.1001/jamanetworkopen.2024.11656)
Supplement: Supplement 2. — Data Sharing Statement [file jamanetwopen-e2411656-s002.pdf]

Data Sharing Statement

Sumibcay. Racial and Ethnic Disparities in COVID-19 Mortality. *JAMA Netw Open*. Published May 21, 2024. doi:10.1001/jamanetworkopen.2024.11656

Data
Data available: No

Additional Information
Explanation for why data not available: Data used in the study is publicly available from the CDC National Center for Health Statistics.
